# Integrated analyses identify MAN1B1 as a candidate regulator of chemotherapeutic tolerance and immunosuppression in bladder cancer

**DOI:** 10.3389/fimmu.2026.1958628

**Published:** 2026-10-07

**Authors:** Mingzhe Jiang, Dong Zhao, Liang Zhang, Dantong Zhu, Jia Li, Zhendong Zheng

**Affiliations:** Department of Oncology, General Hospital of Northern Theater Command, Shenyang, China

**Keywords:** bladder cancer, gemcitabine tolerance, immune evasion, MAN1B1, metabolic rewiring, PI3K-AKT-mTOR axis, single-cell transcriptomics

## Abstract

**Background:**

Gemcitabine (GEM) resistance limits treatment efficacy in bladder cancer (BC). This study identified MAN1B1 as a key driving factor for chemotherapy resistance and malignant progression in BC.

**Methods:**

Bulk-RNA datasets GSE190636 and single-cell RNA-seq dataset GSE250523 from GEO. Use limma package to identify differentially expressed genes (DEGs) in the process of GEM resistance, and then perform GO and KEGG enrichment analysis. Screening candidate targets through molecular docking with GEM, and further evaluation through molecular dynamics simulation and GEPIA survival analysis. GSEA analysis is used to explore pathways associated with key genes. Perform single-cell analysis using Seurat, SingleR, Monocle 2, CellChat, and scTenifoldKnk, including cell annotation, pseudo time trajectory, intercellular communication, and virtual knockout. Carry out functional experiments in bladder cancer cell lines, including cell viability, wound healing Transwell, Colony formation and Western blot analysis.

**Results:**

Through comprehensive transcriptome analysis and single-cell sequencing, we found that elevated levels of MAN1B1 are associated with poorer clinical outcomes. Computational molecular docking and molecular dynamics simulations indicate the potential binding interaction between MAN1B1 and GEM.This enzyme orchestrates a dual microenvironmental reprogramming by hyperactivating metabolic cascades while simultaneously dampening anti-tumor immune surveillance, predominantly originating from the epithelial compartment to modulate intercellular crosstalk. Functional disturbance experiments showed that knockout of MAN1B1 was associated with decreased cell survival, migration, and invasion, as well as increased sensitivity to GEM, while ectopic expression of MAN1B1 increased cell survival and was associated with enhanced PI3K/AKT/mTOR pathway activity.

**Conclusions:**

Our findings uncover the multifaceted regulatory network governing MAN1B1, establishing its utility as an innovative prognostic indicator and an actionable therapeutic vulnerability for overcoming GEM insensitivity in urological oncology.

## Introduction

Bladder cancer (BC) is the most common tumor that affects the male genitourinary tract. It accounts for a significant proportion of cancer-related mortality around the world (1–3). Recently, its incidence and mortality have increased significantly, bringing a huge pressure to family and society (4–6). This disease (also called BLCA) shows notable clinical heterogeneity. According to the depth of bladder wall invasion, it can be generally divided into muscle-invasive (MIBC) or non-muscle-invasive (NMIBC) subtypes (7). With aggressive biology, MIBC progresses rapidly and exhibits a tendency for early metastasis, leading to high rates of recurrence and death (8). In about 80% of newly diagnosed BC cases, the disease presents as NMIBC, which is linked to a good five-year survival prognosis (9). However, most of these persons experience recurrence of the disease within five years, and approximately half evolve into MIBC or develop distant metastases (10). Currently, immunotherapy, systemic or intravesical chemotherapy, surgical resection, local instillation therapies, antibody–drug conjugates, and targeted agents are conventional treatment approaches for BC. Tumor stage and grade mainly determine the selection of intervention. Meanwhile, therapeutic approaches are designed according to the exact disease stage (11). In general, standard management for low-grade, stage Ta lesions (non-invasive papillary urothelial carcinoma) consists of intravesical chemotherapy with gemcitabine or mitomycin. For high-grade Ta tumors, transurethral resection of the bladder tumor (TURBT) is required (12). Furthermore, patients need to receive three-year bacillus Calmette-Guérin (BCG) intravesical immunotherapy. Certainly, intravesical chemotherapy is still an option (12, 13). To manage T1 NMIBC (infiltration of tumor into the lamina propria), repeat TURBT is not enough. It is also essential to conduct subsequent BCG for low-grade cases. For high-grade T1 disease, it is generally recommended to adopt radical cystectomy (12). Typically, neoadjuvant or adjuvant chemotherapy is combined with surgical resection to care for MIBC. Nonetheless, chemoresistance frequently develops in advanced or resectable disease, leading to unfavorable survival results, metastatic spread, and tumor recurrence. Thus, in BLCA management, a key challenge is how to address therapeutic resistance (14, 15).

As a foundation of BC treatment, chemotherapy provides a pronounced survival benefit and functions as a very prospective therapeutic method. Gemcitabine (GEM) is a deoxycytidine analog with effective antineoplastic property, which has been extensively applied to deal with various solid tumors (16). In terms of its mechanism, it can terminate DNA chain elongation to inhibit tumor cell proliferation, cause cell death, and effectively manage bladder cancer (17, 18). For NMIBC, GEM is given through intravesical instillation, which constitutes a part of holistic regimens for MIBC (19, 20). Although GEM is regarded as a first-line agent, it has limited therapeutic impact on patients. In NMIBC, GEM therapy is only clinically beneficial to about 50% of patients (21). For MIBC, it can only suppress or reverse disease progression in a limited manner. The objective response rate to systemic GEM seldom surpasses 40%, in which only a few cases show durable responses (22, 23). At the same time, there are some challenges inhibiting GEM therapy. Specifically, it cannot selectively target malignant cells, which will trigger reduced response rates and increased recurrence over prolonged treatment courses. Worse still, significant systemic toxicity will occur and chemoresistance develops. All these factors jointly improve the risk of adverse events. Owing to these restrictions, GEM cannot realize wide application in BC. Therefore, it is necessary to thoroughly investigate the molecular basis of GEM resistance in this malignant disease (24–26). By using transcriptomic datasets (GSE190636 and TCGA-BLCA) from the GEO and TCGA repositories, this study has identified genes related to GEM resistance, described the sequential acquisition of chemoresistance by cancer cells, and uncovered novel therapeutic targets.

## Methods

### Data acquisition

Based on the public GEO archive, this study retrieved transcriptomic profiles (accession GSE190636) that contained sixteen specimens. In this collection, transcriptional changes within T24 urothelial carcinoma lineages during the evolution of chemoresistance across designated temporal intervals (zero, three, seven, plus fifteen days) were supervised. In every stage, quadruplicate biological measurements were conducted. From the same archive, this study obtained single-cell resolution transcriptomes (GSE250523) covering healthy and malignant bladder tissues.

### Differential expression profiling

The limma computational framework was harnessed for temporal comparisons. The purpose was to identify transcriptionally altered loci. Comparisons were conducted between baseline and day three, day three and day seven, as well as day seven and day fifteen. Thresholds of absolute log2 fold changes greater than 0 and adjusted P < 0.05 were taken as the criteria for statistical significance.

### Functional pathway mapping

To examine biological significance of the identified transcripts, this study employed pathway mapping. To be specific, the clusterProfiler context explained hidden molecular cascades by promoting KEGG database querying and Gene Ontology categorization.

### Computational ligand docking

This study implemented computational docking protocols to assess physical associations that connected the translated gene products and chemotherapeutic agent. At first, respective polypeptide accession codes were extracted by mapping candidate identifiers via the UniProt repository. Subsequently, every chosen macromolecule’s three-dimensional conformational models were acquired from the AlphaFold resource. In this way, strong spatial coordinates were established. Beyond that, the ZINC platform was adopted to get the stereochemical architecture of ligand. Before simulation, apart from stripping aqueous molecules, PyMOL utilities inserted polar hydrogens and excised native cocrystallized compounds to improve macromolecular geometries. Consequently, the biochemical milieu was optimized. For ensuring sufficient spatial sampling, this study used AutoDockTools (version 1.5.6) to generate grid boxes containing dimensions of forty units cubed and potential interaction zones. Additionally, AutoDock Vina (release 1.1.2) was leveraged to quantify preferred orientations and binding affinities. The pose with minimal thermodynamic free energy was determined as the premier interaction model. For complexes whose spontaneous interaction energies were more negative than negative seven kilocalories per mole, they were categorized as having strong binding potential. Lastly, predicted poses were matched against empirical crystalline ligand orientations to confirm structural validity. On this basis, computational reliability was validated.

### Molecular dynamics evaluations

This study used Gromacs2022 suite for dynamic behavioral profiling. Regarding macromolecules, the AMBER14SB parameter set solvated in a TIP3P aqueous medium was adopted. Furthermore, the GAFF topologies were employed by chemotherapeutic compound. These two entities were incorporated into a consistent periodic simulation box, which was sustained under isothermal-isobaric limitations. During the whole temporal evolution, the LINCS methodology was taken to restrict covalent hydrogen linkages. A two-femtosecond temporal increment was adopted. Based on the Particle Mesh Ewald method, Long-range Coulombic forces were calculated using a distance threshold of 1.2 nanometers. On the contrary, van der Waals contacts were limited to a ten-angstrom range and updated once every ten iterations. For thermal regulation at 298 Kelvin, it depended on V-rescale coupling. Meanwhile, Berendsen barostatting was used for one-bar pressure maintenance. After temporary hundred-picosecond equilibration stages (NVT plus NPT ensembles), a holistic hundred-nanosecond production run tracked system dynamics, with coordinate snapshots decapicosecond intervals being archived. In post-trajectory evaluations, VMD and PyMOL are used for visual inspection. Through g_mmpbsa utility, thermodynamic affinities that connected the pharmacological agent to its macromolecular receptor were quantified.

### Prognostic biomarker interrogation

Through the GEPIA web portal (an aggregator combining GTEx plus TCGA transcriptomic cohorts), this study interrogated prognostic meaning of the computationally prioritized candidates. For isolating biomarkers with in-depth prognostic disparities, Kaplan-Meier estimators were produced to compare general patient longevity stratified by transcript abundance.

### Pathway enrichment assessments

Gene Set Enrichment methodologies contrasted pathway activities that differentiated extreme phenotypic cohorts, thereby explaining wider functional outcomes. Biological routes that satisfied false discovery rates below 0.25 and nominal probabilities under 0.05 were considered remarkably overrepresented.

### Single cell data analysis

Firstly, strict quality control was applied to single-cell data from the GSE250523 dataset. Specifically, cells with less than 200 or more than 10000 genes detected, as well as cells with mitochondrial gene expression ratios above 20%, were excluded to remove low-quality cells and potential double cells. After filtering, the NormalizeData function in the Seurat software package is used to standardize the data, and the FindVariable Features function is used to screen for high variable genes. Subsequently, principal component analysis was conducted, and the optimal number of principal components was determined by combining the ElbowPlot and JackStrawPlot functions. Further use the FindClusters function to complete cell clustering, and utilize the RunUMAP function for UMAP dimensionality reduction to achieve visual display of different cell populations. Finally, the FindAllMarkers function was used to identify cluster specific marker genes, and cell type annotation was performed using the SingleR software package to construct a detailed map of the tumor microenvironment.

### Pseudotemporal ordering

To deduce the evolutionary and differentiation dynamics governing diverse cellular subsets, we employed the Monocle 2 algorithm for pseudotemporal ordering. We initially built CellDataset structures leveraging the previously isolated highly variable transcripts, subsequently applying the DDRTree technique for dimensionality reduction. This allowed the reconstruction of cellular developmental paths utilizing the Minimum Spanning Tree (MST) framework. The plot_cell_trajectory utility then generated visual representations of the cellular states alongside their pseudotemporal distributions. Furthermore, we executed branch expression analysis modeling (BEAM) to pinpoint genes specifically associated with distinct lineage bifurcations, integrating these findings with functional enrichment evaluations to uncover the biological processes driving the observed transitional shifts.

### Intercellular crosstalk modeling

We investigated the intercellular signaling crosstalk among the distinct cellular clusters utilizing the CellChat R toolkit. By feeding the normalized expression profiles into this platform, we established a comprehensive intercellular interaction network. The identification of upregulated signaling molecules and their corresponding partnerships relied on the identifyOverExpressedGenes and identifyOverExpressedInteractions commands, respectively. Following this, we applied the computeCommunProb and filterCommunication routines to forecast probable ligand-receptor pairings. Ultimately, the aggregateNet visualization tool mapped the global architecture of these cellular communication pathways.

### In silico gene ablation

In silico gene ablation was executed utilizing the scTenifoldKnk platform (release 1.0.1) to uncover subordinate regulatory cascades influenced by pivotal transcripts. Furthermore, heavily perturbed downstream effectors served as seeds for constructing interactive co-expression webs via the GeneMANIA internet resource.

### Cell culture

Human bladder cancer (BCa) cell lines 5637 and T24 were purchased from the cell bank of the Chinese Academy of Sciences (Shanghai, China). The cells were cultured in DMEM medium (Gibco, Shanghai, China) supplemented with 10% fetal bovine serum (FBS) and 100 U/mL penicillin/streptomycin (Sangon, Shanghai, China), and placed in a constant temperature and humidity incubator at 37°C and 5% CO_2_.

### Metabolic vitality quantification

We evaluated cellular survival rates utilizing a resazurin-based reduction protocol. In brief, we seeded 5,000 cells per well within 96-well formats and allowed them to adhere for a full day. The cultures then received varying doses of the experimental compounds for durations of either twenty-four, forty-eight, or seventy-two hours. Post-exposure, we introduced 10 microliters of resazurin reagent (0.2 mg/mL) into every well, continuing the incubation at 37 degrees Celsius for an additional four hours. A fluorescence microplate detector then quantified the signal, setting the excitation and emission parameters to 560 nm and 615 nm, respectively. We calculated the final survival fractions as relative percentages against the DMSO-treated controls, ensuring all raw readings underwent background correction.

### Scratch assay

The cells’ migratory capabilities were evaluated by a wound healing paradigm. The cultures were grown in six-well formats until near-complete confluence was achieved. Furthermore, a sterile plastic pipette head was used to create an aligned linear wound on the cellular monolayer. Then, phosphate-buffered saline (PBS) was employed to gently wash away the dislodged debris. The rest of cultures were kept in a serum-deprived context. They were incubated under a 5% carbon dioxide atmosphere at 37 degrees Celsius. Images of the wound gap at the initial timepoint, as well as at twenty-four and forty-eight hours post-wounding were captured using an inverted optical microscope. To quantify the migratory progress, this study employed ImageJ software to assess the remaining gap distances. Furthermore, the relative motility was expressed as the wound closure’s percentage relative to the initial baseline width.

### Transwell migration and invasion evaluations

Transwell inserts with 8-micrometer pores were adopted to examine the migratory and invasive behaviors. To evaluate motility, the cells were resuspended in a serum-free solution and 50,000 cells were plated into the upper compartments. As for the bottom reservoirs, they used complete medium containing 10% fetal bovine serum to function as a chemoattractant gradient. During the assessment of invasiveness, before introduction of the cells, a 1:8 diluted Matrigel matrix was followed to pre-treat the upper membrane surfaces. In the wake of an 18 to 24-hour incubation period at 37 degrees Celsius, this study carefully removed the non-translocated cells present on the apical side. On the contrary, 4% paraformaldehyde was used to fix the cells successfully traversing to the basal membrane. Later, 0.1% crystal violet solution was leveraged for staining. Moreover, five distinct microscopic visual fields were randomly chosen per insert for counting the traversed cells.

### Western blot analysis

We extracted total proteomes utilizing RIPA lysis buffer supplemented with both protease and phosphatase inhibitor cocktails. A BCA quantification kit then determined the precise protein concentrations. We resolved equivalent protein aliquots (ranging from 30 to 50 micrograms) via SDS-PAGE electrophoresis before electroblotting them onto polyvinylidene difluoride (PVDF) filters. These membranes were subsequently blocked for one hour at ambient temperature using a 5 percent non-fat dry milk solution prepared in TBST. After blocking, the filters were probed overnight at 4 degrees Celsius with primary antibodies directed against the proteins of interest. Following extensive TBST rinses, we applied horseradish peroxidase-linked secondary antibodies for a one-hour incubation at room temperature. An enhanced chemiluminescence (ECL) substrate facilitated the visualization of the immunoreactive bands, which we then quantified densitometrically using ImageJ. Either GAPDH or β-actin functioned as the reference loading standard.

### Colony formation assay

Based on a standard colony-forming protocol, this study assessed the cell’s clonogenic survival and proliferative capacity. In the wake of enzymatic dissociation, 500-1,000 cells were plated into six-well culture dishes’ each well. To facilitate the emergence of distinct macroscopic colonies, we maintained cultures in complete growth media for 10–14 days. Furthermore, the resulting cellular clusters were fixed for fifteen minutes using 4% paraformaldehyde. Moreover, staining was performed with 0.1% crystal violet over a 30-minute period. After rinsing with PBS, a brightfield microscope was harnessed to count all visible colonies with a diameter of more than 50 micrometers. The final colony count was divided by the original number of plated cells to calculate clonogenic efficiency.

### Statistical analysis

In the R programming environment (version 4.1.1), all statistical assessments were carried out. Specifically, two independent cohorts’ disparities were measured using the Wilcoxon rank-sum procedure. During the Kruskal-Wallis test, multiple groups were compared. For the *in vitro* experimental data, statistical modeling was conducted via GraphPad Prism 9, in which paired or unpaired groups’ pronounced variations were identified through the Student’s t-test. A probability value below the 0.05 threshold was deemed statistically significant.

## Results

### Identification of differentially expressed gene

To explore genes with dynamic expression changes during the acquisition of gemcitabine resistance, this study analyzed the GSE190636 transcriptomic dataset available in the GEO repository. In addition, strict standards—|Log_2_FC| > 0 and adjusted *P* < 0.05 were employed to identify differentially expressed genes (DEGs) in sequential time intervals (days 0 versus 3, 3 versus 7, and 7 versus 15). For downstream integrative analyses, this study retained downregulated and upregulated transcripts from these pairwise comparisons (**Figure 1**). Beyond that, comprehensive functional enrichment assessments were conducted to interpret the biological meaning of bladder cancer’s transcriptional changes. As demonstrated by Gene Ontology (GO) analysis, DEGs were significantly overrepresented in structures and processes associated with the leading edge of migrating cells, focal adhesions, collagen-rich extracellular matrix elements, actin cytoskeleton binding, and wider extracellular matrix organization (**Figure 2A**). As highlighted by Kyoto Encyclopedia of Genes and Genomes (KEGG) pathway mapping, there was a remarkable relationship with regulatory components of the Hippo signaling cascade (**Figure 2B**). That is to say, it may involve in the chemoresistance’s evolution.

**Figure 1:**
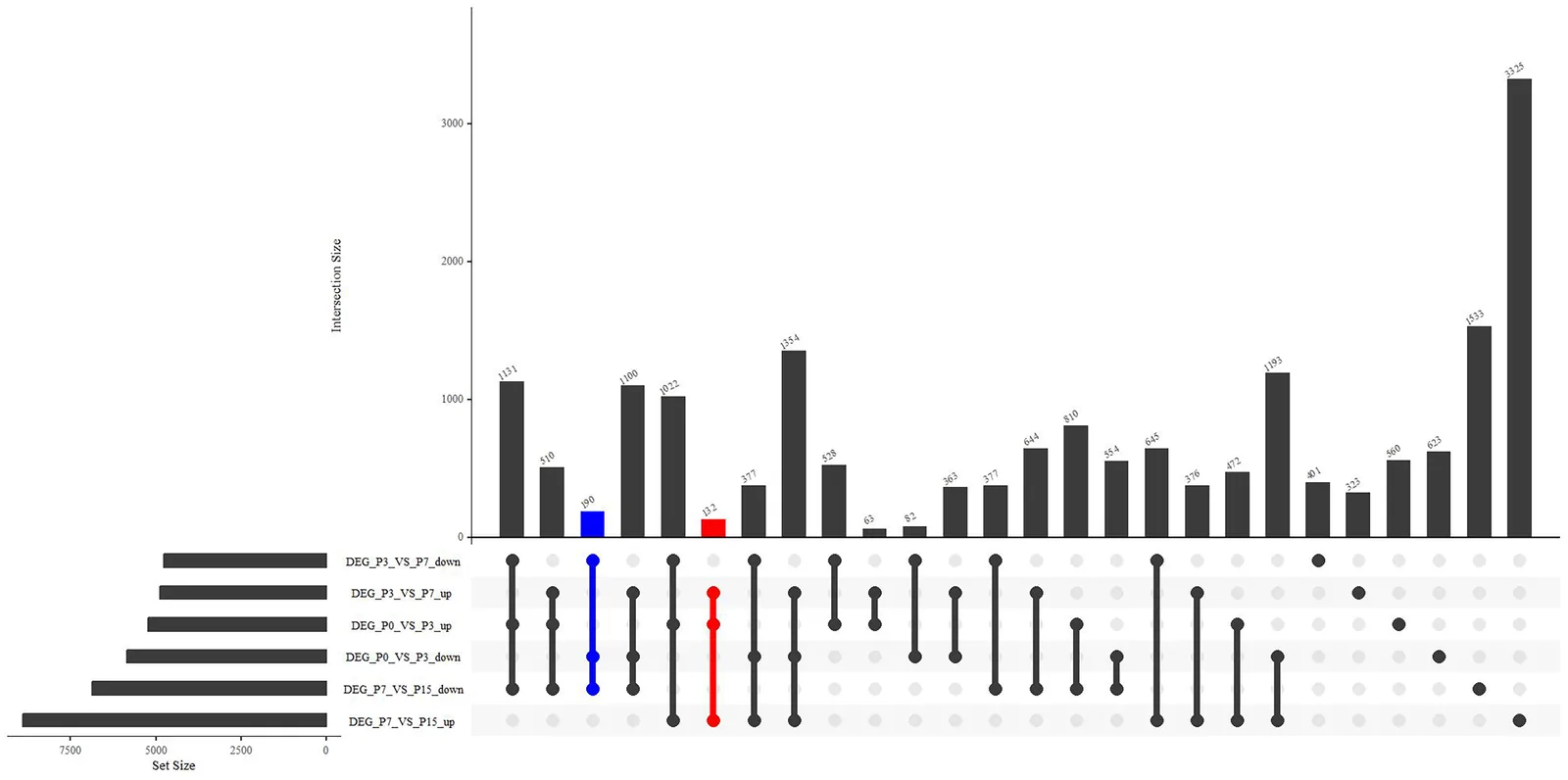
Identification of differentially expressed genes.

**Figure 2:**
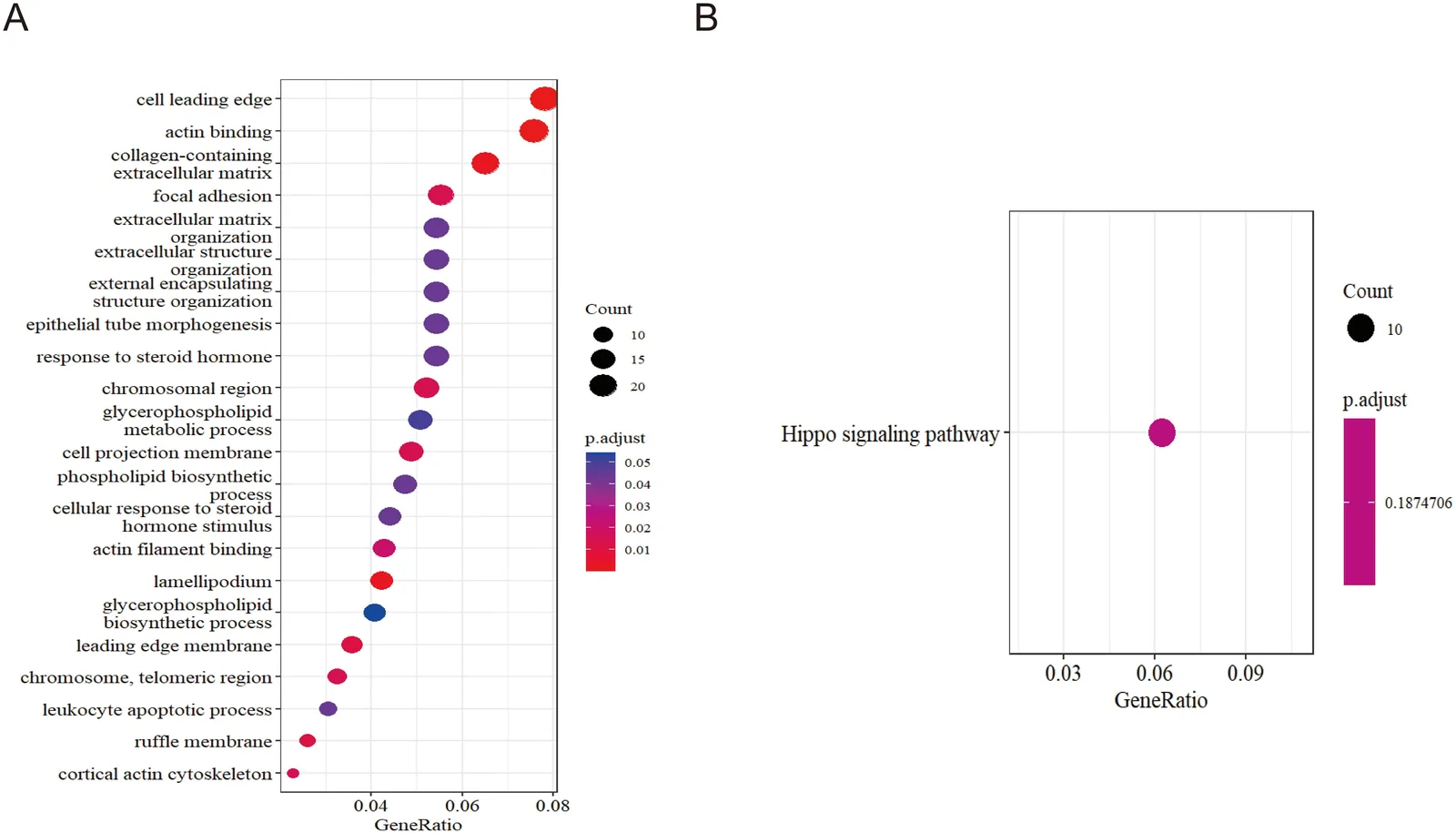
Functional annotation analysis of DEGs. **(A)** GO analysis; **(B)** KEGG analysis.

### Molecular docking analysis

Based on the UniProt database, this study systematically mapped all identified DEGs to their corresponding protein products. Additionally, the AlphaFold structural repository was harnessed to retrieve high-confidence three-dimensional models for 1,641 distinct protein entities. On this basis, a powerful structural framework was built for interaction screening. Moreover, chemical fidelity was ensured by sourcing gemcitabine’s three-dimensional conformation from the ZINC15 database. AutoDock Vina was used to implement molecular docking simulations between each protein structure and gemcitabine. For each target, the conformation that generated the lowest predicted binding free energy was chosen as the best pose. This study applied a dual filter - binding affinity < −7.0 kcal/mol and concordant upregulation in resistant cells - to prioritize a subset of high-confidence interactors. Notably, the protein encoded by UniProt ID P53396 showed an unusually strong interaction, in which a calculated binding energy was −8.5 kcal/mol (**Table 1**). Other high-affinity binders involved EIF3M, KEAP1, ACLY, MAN1B1, ADAM8, KIF20A, EML2, PMP22, and RIMKLB, indicating that they may mediate mechanism or resistance development of gemcitabine. Overall, this systematic docking pipeline depicted a pattern of potential molecular interfaces between gemcitabine and resistance-related proteins. This laid a foundation for reasonable combination tactics and mechanistic exploration.

**Table 1:** The result of molecular docking.

No.	Entry	Affinity.kcal.mol.	From	Protein.names
1	A0A024R1T9	-8.2	ACLY	ATP-citrate synthase (EC 2.3.3.8) (ATP-citrate (pro-S-)-lyase) (Citrate cleavage enzyme)
2	A0A024R1Y2	-8.3	ACLY	ATP-citrate synthase (EC 2.3.3.8) (ATP-citrate (pro-S-)-lyase) (Citrate cleavage enzyme)
3	A0A024R7C0	-8	KEAP1	Kelch-like ECH-associated protein 1
4	E7ESM3	-8.1	EIF3M	Eukaryotic translation initiation factor 3 subunit M
5	H0YI64	-8	MAN1B1	alpha-1,2-Mannosidase (EC 3.2.1.-)
6	K7EIK7	-8	EML2	EMAP like 2
7	O95235	-8.1	KIF20A	Kinesin-like protein KIF20A (GG10_2) (Mitotic kinesin-like protein 2) (MKlp2) (Rab6-interacting kinesin-like protein) (Rabkinesin-6)
8	P53396	-8.5	ACLY	ATP-citrate synthase (EC 2.3.3.8) (ATP-citrate (pro-S-)-lyase) (ACL) (Citrate cleavage enzyme)
9	P78325	-8.1	ADAM8	Disintegrin and metalloproteinase domain-containing protein 8 (ADAM 8) (EC 3.4.24.-) (Cell surface antigen MS2) (CD antigen CD156a)
10	Q14145	-8.1	KEAP1	Kelch-like ECH-associated protein 1 (Cytosolic inhibitor of Nrf2) (INrf2) (Kelch-like protein 19)
11	Q9NR77	-8	PMP22	Peroxisomal membrane protein 2 (22 kDa peroxisomal membrane protein)
12	Q9ULI2	-8.1	RIMKLB	Beta-citrylglutamate synthase B (EC 6.3.1.17) (N-acetyl-aspartylglutamate synthetase B) (NAAG synthetase B) (NAAGS) (EC 6.3.2.41) (Ribosomal protein S6 modification-like protein B)

### Survival analysis of candidate targets

In combination with TCGA-BLCA patient data, survival analysis was conducted based on the GEPIA platform to assess these candidate interactors’ clinical relevance. As indicated by Kaplan–Meier curves, higher expression levels of *ACLY*, *KIF20A*, and *PMP22* were associated with a trend towards poorer overall survival, yet these associations were not statistically significant (**Figures 3A–C**). On the contrary, high expression levels of *ADAM8*, *EIF3M*, and *KEAP1* were related to elevated survival results, which showed no statistical significance. Compared to patients with high expression, those with low *MAN1B1* expression presented significantly reinforced survival (*P* = 0.00022; **Figures 3D–G**). Due to this strong prognostic signal, *MAN1B1* was chosen for holistic functional and mechanistic follow-up.

**Figure 3:**
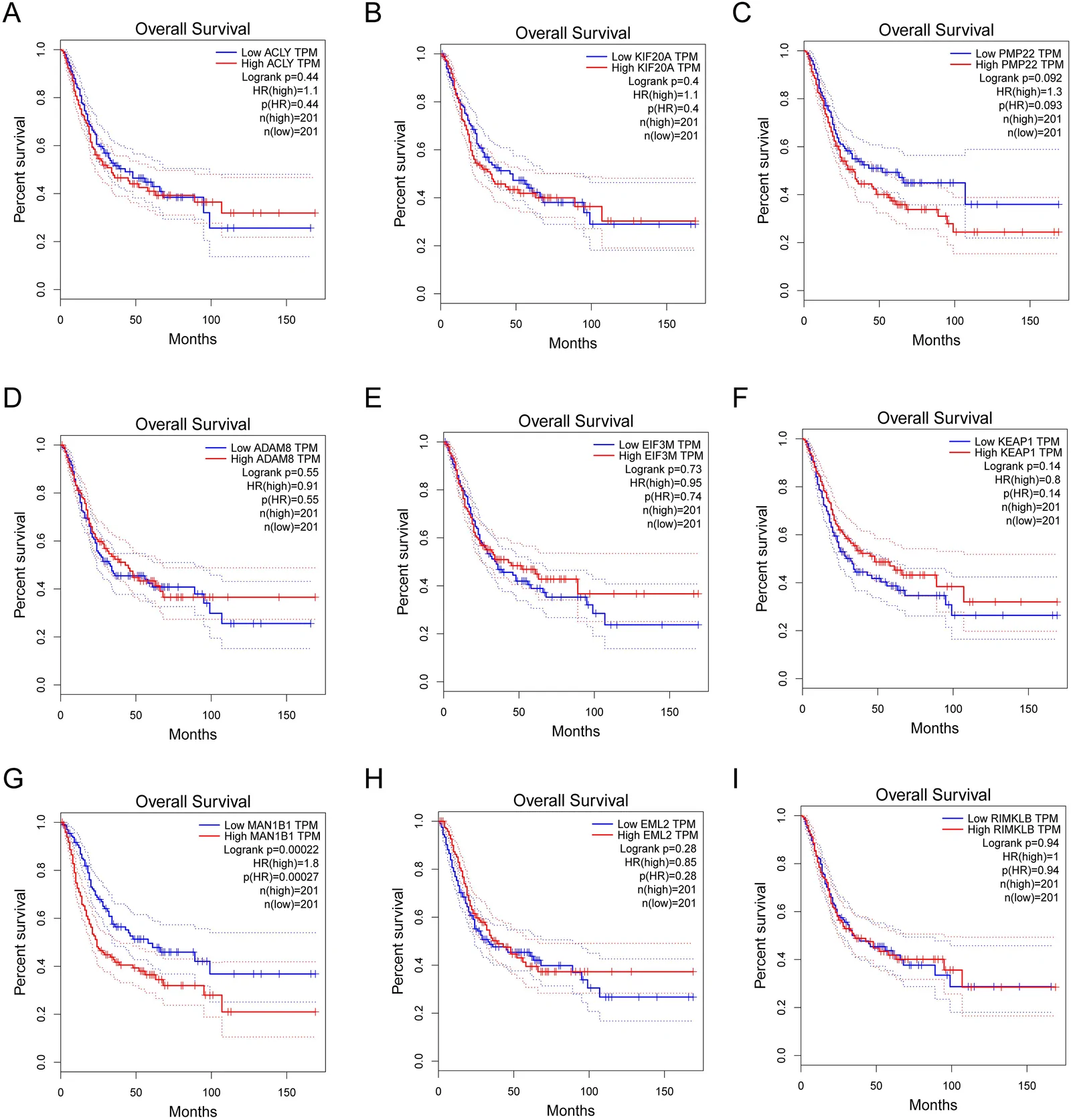
Survival analysis of 9 potential targets. **(A)** ACLY; **(B)** KIF20A; **(C)** PMP22; **(D)** ADAM8; **(E)** EIF3M; **(F)** KEAP1; **(G)** MAN1B1; **(H)** EML2; **(I)** RIMKLB.

### Molecular dynamics simulation

In this study, 100-nanosecond all-atom molecular dynamics (MD) simulation of the MAN1B1–gemcitabine complex was performed to verify and improve the static docking predictions. As revealed by Root Mean Square Deviation (RMSD) trajectories, the complex became initially stable within the first 60 ns. Between 60–100 ns, there was a moderate fluctuation, manifesting ligand repositioning in the binding pocket (**Figure 4A**). After 60 ns, the distance between gemcitabine and both the defined binding site and the protein center of mass got stable (**Figure 4D**). During the whole simulation, the Radius of Gyration (Rg) was still constant (**Figure 4B**), suggesting preserved global compactness. Likewise, all residues’ Root Mean Square Fluctuation (RMSF) values were lower than 0.40 nm (**Figure 4C**), highlighting structural rigidity. Initial stabilization of Buried Solvent Accessible Surface Area (Buried SASA) demonstrated stable interfacial contact (**Figure 4E**). Although conformational overlays showed transient dispersion (**Figure 4F**), electrostatic (ELE) and van der Waals (VDW) interaction energies were still stable in the first 60 ns (**Figure 4G**). GLU-601 and GLU-627 belonged to key interacting residues (**Figure 4H**). Over time, hydrogen bond occupancy fluctuated between one and three bonds (**Figure 4I**), in which THR-626, ARG-535, and GLU-537 mediated specific interactions. ANS-625, GLU-540, and PHE-267 formed Van der Waals contacts (**Figure 4J**). Through these dynamic analyses, a stable, energetically favorable interaction between MAN1B1 and gemcitabine is confirmed, offering a structural rationale for its functional role.

**Figure 4:**
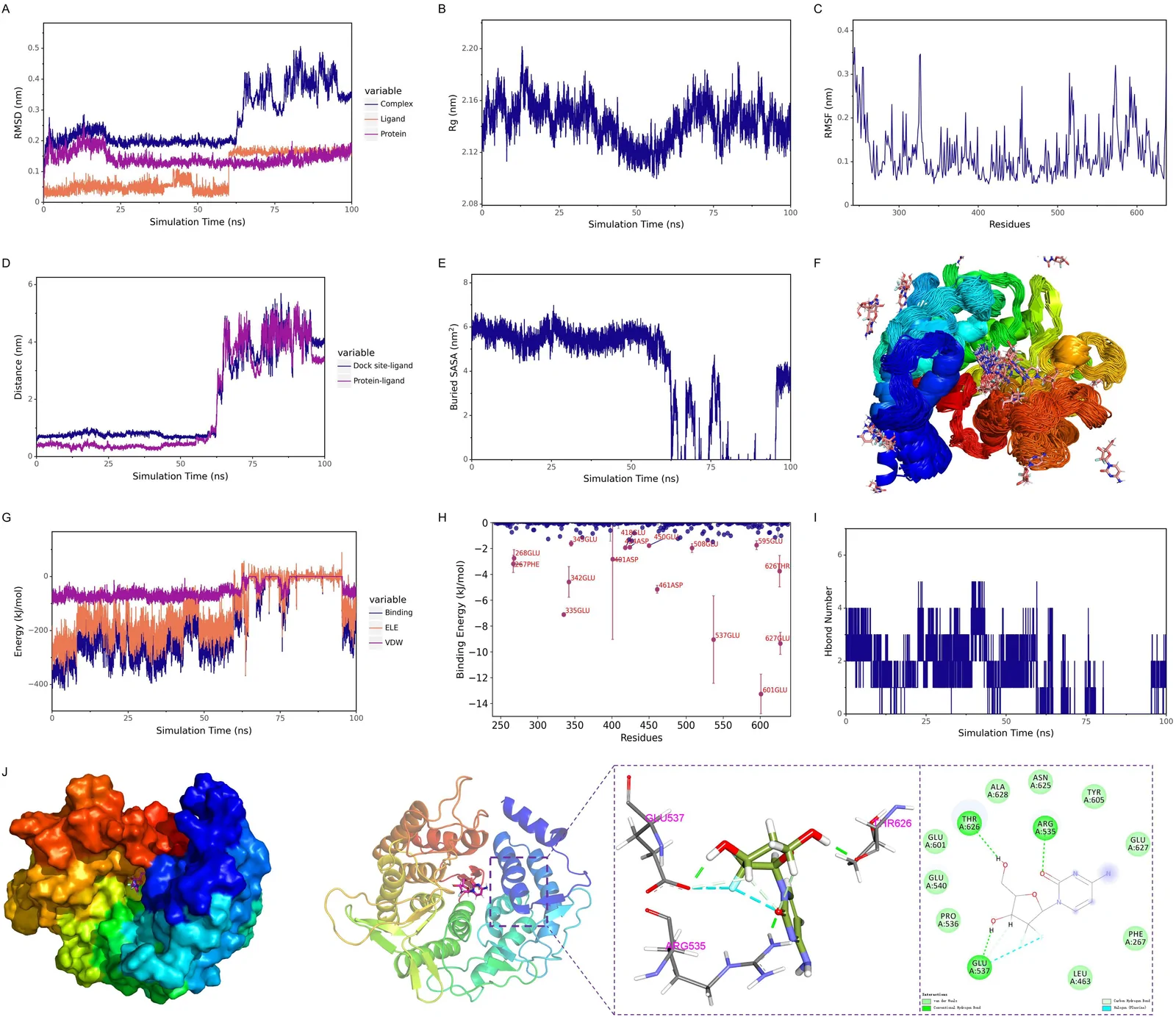
MD results. **(A)** RMSD of complexes, proteins, and small molecule ligand Ligand; **(B)** Rg of complex; **(C)** RMSF of protein in complex; **(D)** The distance between protein and small molecule binding sites; **(E)** Embedded surface area between small molecules and proteins; **(F)** Simulate conformational superposition; **(G)** The binding energy between small molecules and proteins VDW and ELE; **(H)** Contribution of amino acid binding energy; **(I)** Hbond number; **(J)** The interaction between proteins and small molecules.

### GSEA of MAN1B1 in bladder cancer

Single-gene Gene Set Enrichment Analysis (GSEA) was conducted on TCGA-BLCA data to examine the wider biological context of *MAN1B1* activity. High *MAN1B1* expression was remarkably correlated with the activation of many metabolic programs, such as fatty acid catabolism, xenobiotic biotransformation, energy metabolism, and hormone/vitamin processing pathways. However, immune-related signatures (e.g. IgA-producing intestinal immune networks, NOD-like receptor signaling, natural killer cell-mediated cytotoxicity, as well as antigen processing and presentation) were greatly inhibited in the *MAN1B1*-high cohort (**Figure 5**). As indicated by this dual pattern, *MAN1B1* may propel tumor progression via concurrent immune evasion and metabolic rewiring.

**Figure 5:**
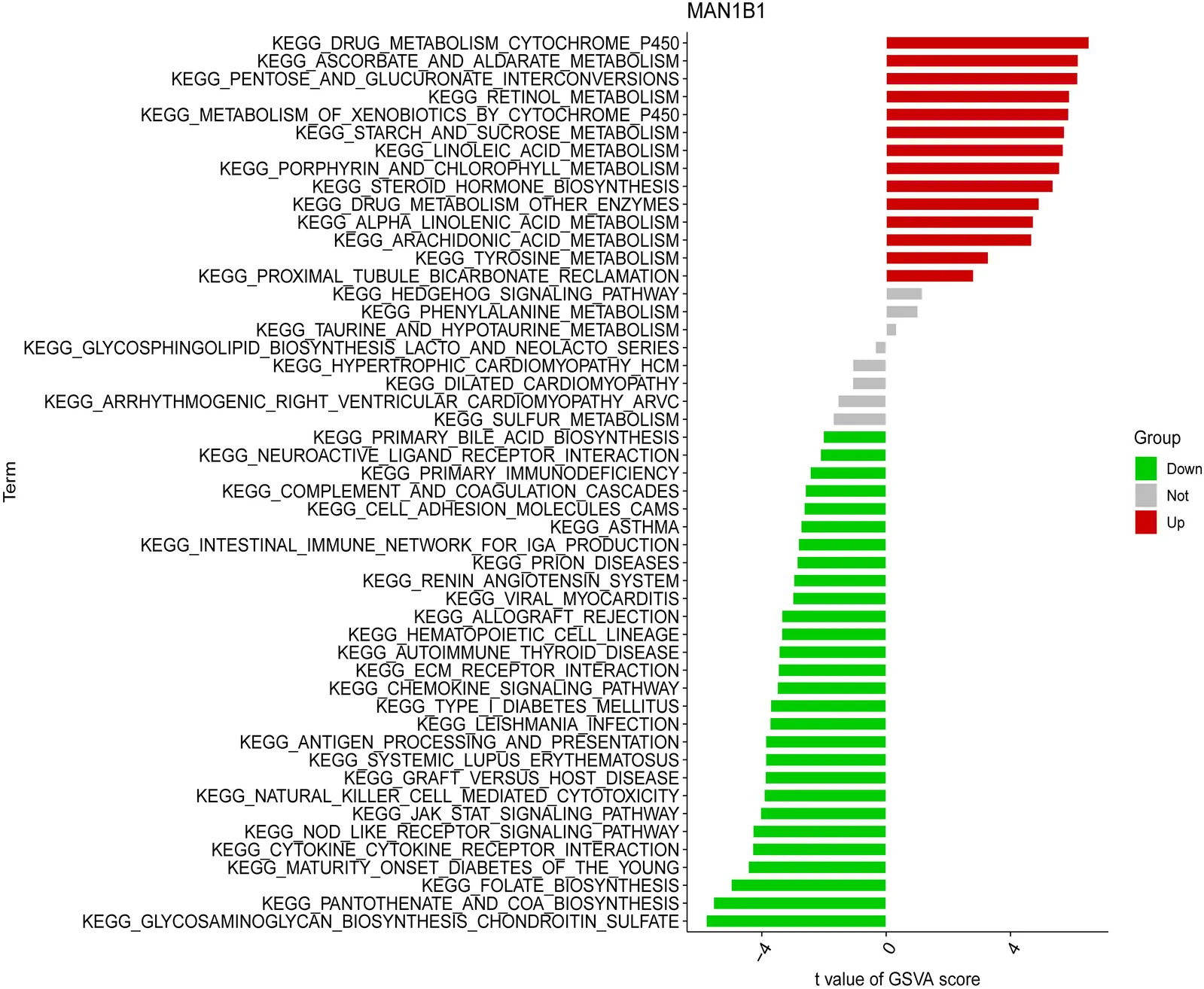
GSEA analysis of MAN1B1.

### Dynamic expression of MAN1B1 across bladder cancer trajectories

Cellular-resolution mapping of *MAN1B1* was realized based on single-cell RNA sequencing (scRNA-seq) analysis of the GSE250523 dataset. Unsupervised clustering and annotation based on canonical markers addressed nine different cell populations: macrophages, B cells, CMPs, T cells, epithelial cells, smooth muscle cells, neutrophils, monocytes, and endothelial cells (**Figure 6A**). *MAN1B1* expression was mainly localized in the epithelial compartment (**Figure 6B**). Pseudotemporal ordering via Monocle 2 rebuilt a persistent differentiation trajectory consisting of three developmental states, which progressed left to right (**Figures 6C, D**). Endothelial cells had enrichment in the earliest branch (**Figure 6E**), whereas *MAN1B1* expression progressively declined along the pseudotime axis (**Figure 6F**), indicating that it was related to an early, potentially adaptive or highly active malignant state.

**Figure 6:**
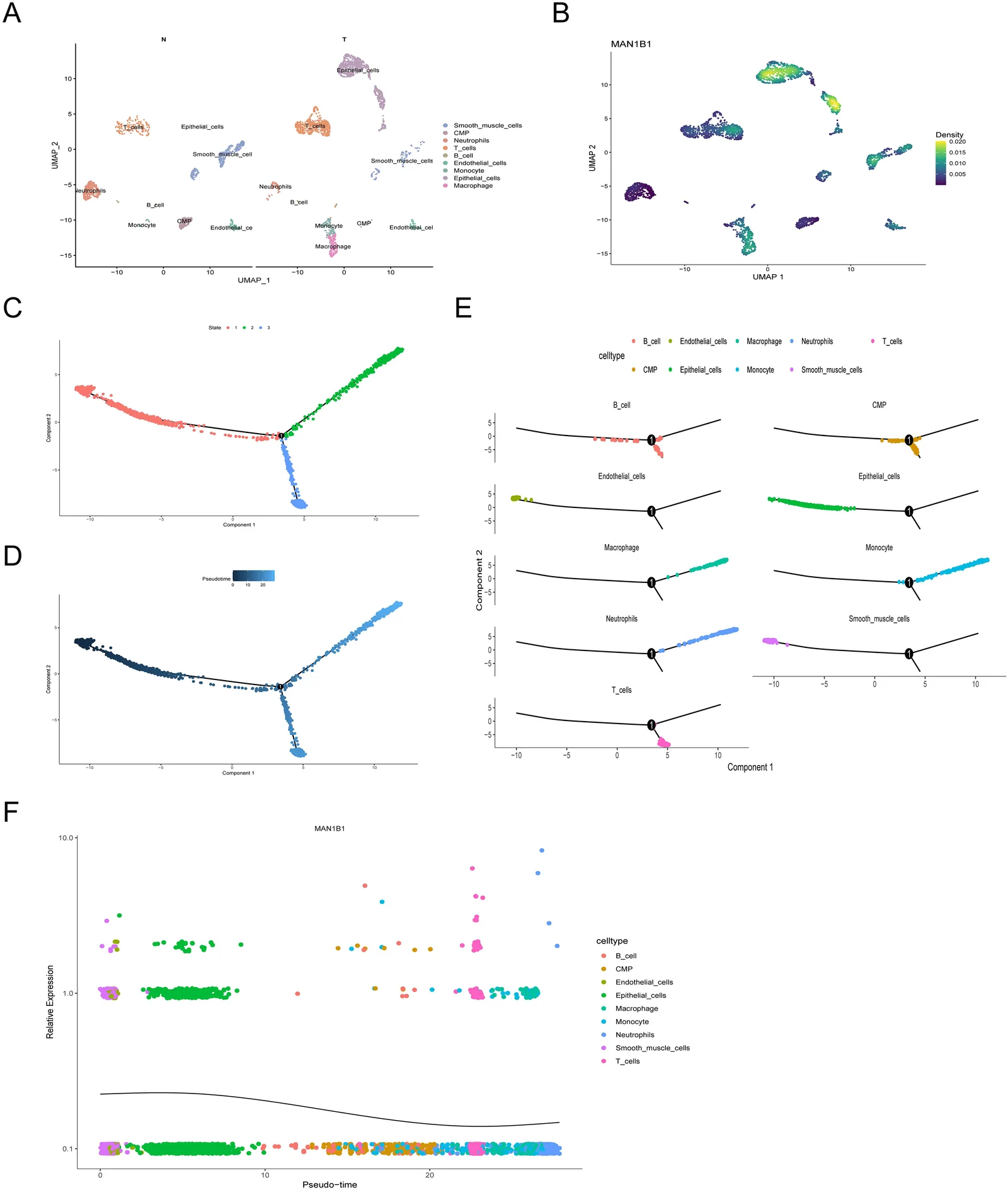
Dynamic characteristics of MAN1B1 expression in the development track of BC. **(A)** UMAP visualization of single cells in normal and BC samples. **(B)** UMAP map of MAN1B1 expression. **(C)** The cell trajectory state diagram displays the developmental trajectory of cells at different pseudo time points, with different cell states displayed in different colors. **(D)** The pseudo time chart simulates the sequence of cell differentiation. **(E)** Pseudo temporal developmental trajectories of all subgroups. **(F)** The relative expression of MAN1B1 at different pseudo times.

### Cell–cell communication mediated by MAN1B1+ epithelial cells

Epithelial cells were stratified into *MAN1B1*^+^ and *MAN1B1*^-^ subsets for investigating their communicative roles. As demonstrated by CellChat analysis, *MAN1B1*^+^ epithelial cells engaged remarkably in signaling via the AMPK pathways, Hippo, and ECM–receptor interaction (**Figure 7A**). Given their remarkably improved communication strength with smooth muscle cells, endothelial cells, and macrophages, they became central hubs in the tumor microenvironmental crosstalk network (**Figure 7B**). As uncovered Ligand–receptor mapping, ECM–receptor interactions supported most of these intercellular dialogues (**Figure 7C**), strengthening the connection between *MAN1B1* and extracellular matrix remodeling.

**Figure 7:**
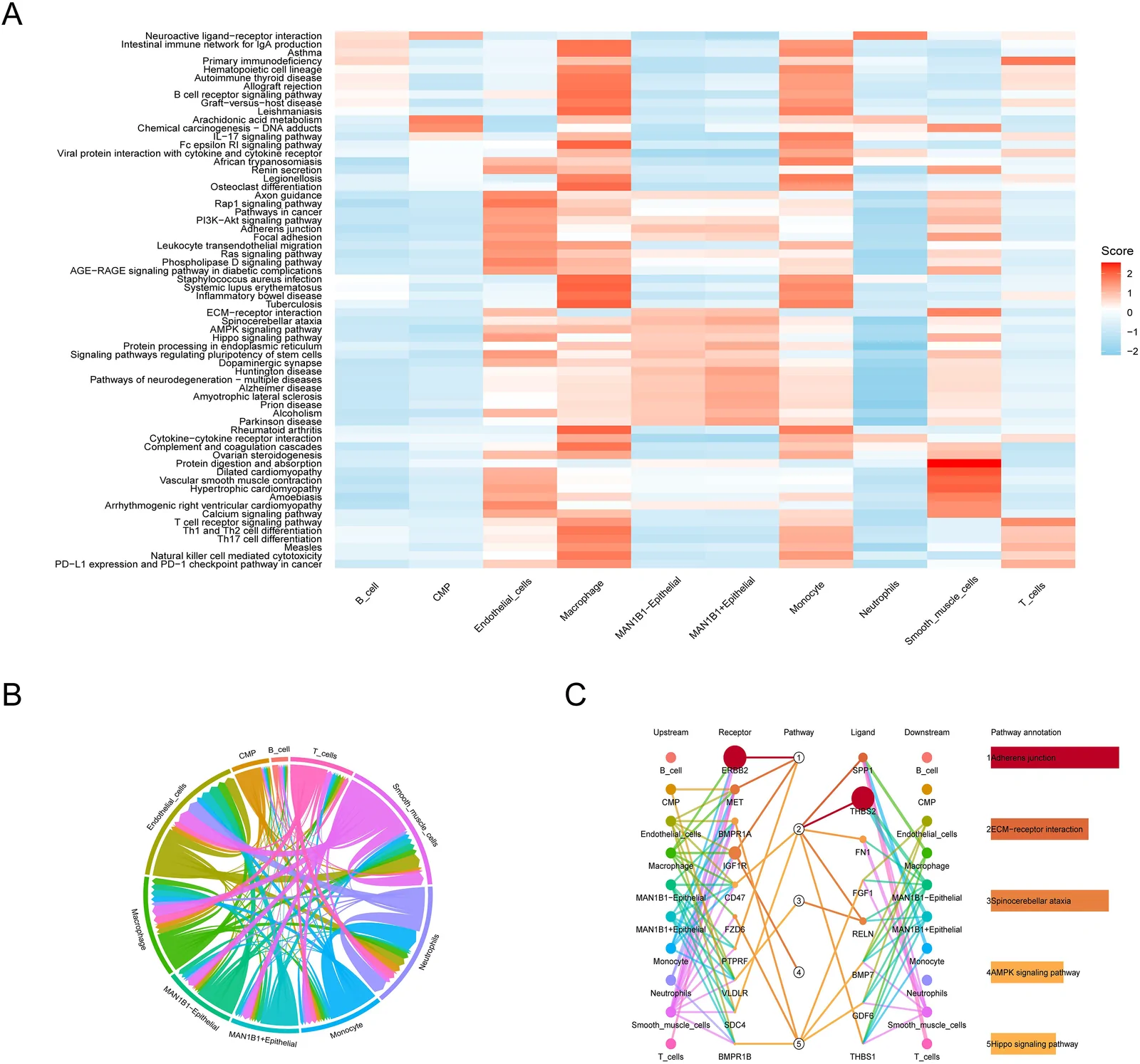
Cell Communication Analysis. **(A)** The interaction between MAN1B1+Epithelial cells and ECM receptors is significantly correlated with pathways such as Hippo signaling pathway and AMPK signaling pathway. **(B)** The string plot shows connections between MAN1B1+Epithelial cells and multicellular cells. **(C)** The intercellular communication chain mediated by the pathway of MAN1B1+Epithelial cells in tumor tissue.

### In silico knockout of MAN1B1 reveals immune-modulatory downstream networks

By using scTenifoldKnk for virtual perturbation of *MAN1B1*, this study found that *TRAF3IP3* and *TAGAP* were the most significantly dysregulated downstream effectors (**Figures 8A, B**). Functional annotation was performed on a co-expression network centered on these genes, which was built via GeneMANIA (**Figure 8C**). GO enrichment underscored immune-centric processes: lymphocyte differentiation/proliferation, leukocyte-mediated immunity, positive regulation of cytokine production, T cell receptor signaling, and antigen receptor-mediated activation. At the subcellular level, these genes gathered together at immune synapses, in which molecular functions involved GTPase binding and TNF receptor superfamily interactions (**Figure 8D**). According to KEGG analysis, enrichment was observed in FcγR-mediated phagocytosis, FcϵRI and NF-κB signaling, innate (RIG-I-like receptor) and adaptive (B and T cell receptor) immune pathways, and leukocyte transendothelial migration (**Figure 8E**). As highlighted by integrated signature, *MAN1B1* plays a potential role in shaping an immunosuppressive niche.

**Figure 8:**
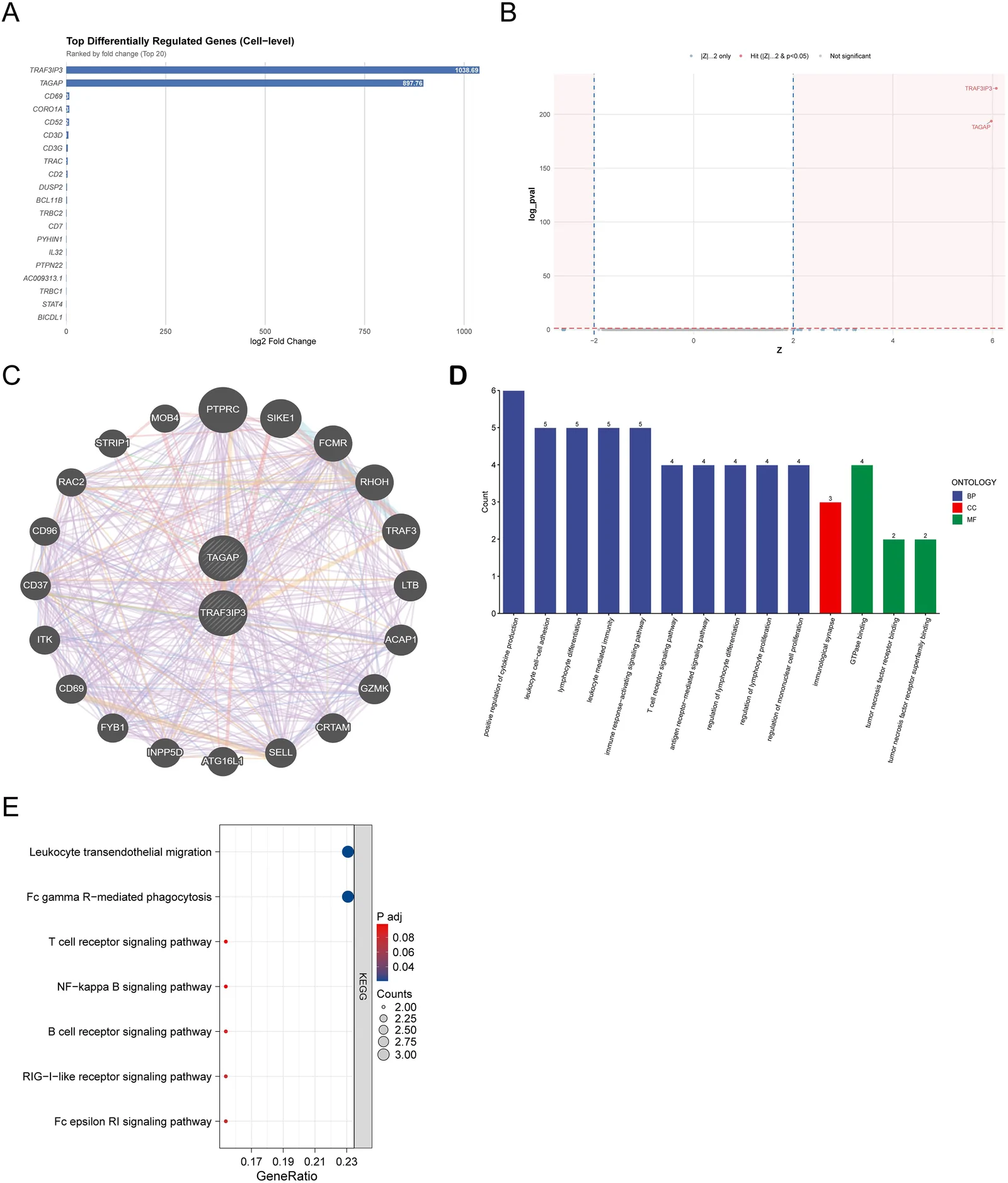
Knockout of MAN1B1 can identify downstream regulatory networks. The computer knockout of MAN1B1 in single cells **(A, B)** revealed the top ranked differentially disordered genes. A: Bar chart; B: Scatter plot. **(C)** The co expression network of TRAF3IP3 and TAGAP. **(D)** GO enrichment analysis. **(E)** KEGG analysis.

### Functional validation of MAN1B1 in bladder cancer cell lines

Through experiment, oncogenic role of *MAN1B1* in T24 and 5637 bladder cancer lines was verified. Overexpression of *MAN1B1* remarkably reinforced cellular viability, while siRNA-mediated knockdown (si-MAN1B1#1 and #2) greatly decreased it (**Figure 9A**). Besides, *MAN1B1* silencing could effectively inhibit both invasive and migratory abilities (**Figures 9B, C**). As revealed by Western blotting, *MAN1B1* overexpression upregulated critical effectors of the PI3K/AKT/mTOR axis (i.e. PI3K, AKT, p-AKT, mTOR, p-mTOR, and the anti-apoptotic protein Bcl-2), but downregulated pro-apoptotic Caspase-3 and Bax (**Figure 9D**). Notably, *MAN1B1* knockdown sensitized cells to gemcitabine, with decreased colony formation and strengthened cytotoxicity being evidence (**Figure 9E**). All in all, *MAN1B1* disturbance is associated with changes in protein expression related to the PI3K/AKT/mTOR pathway.

**Figure 9:**
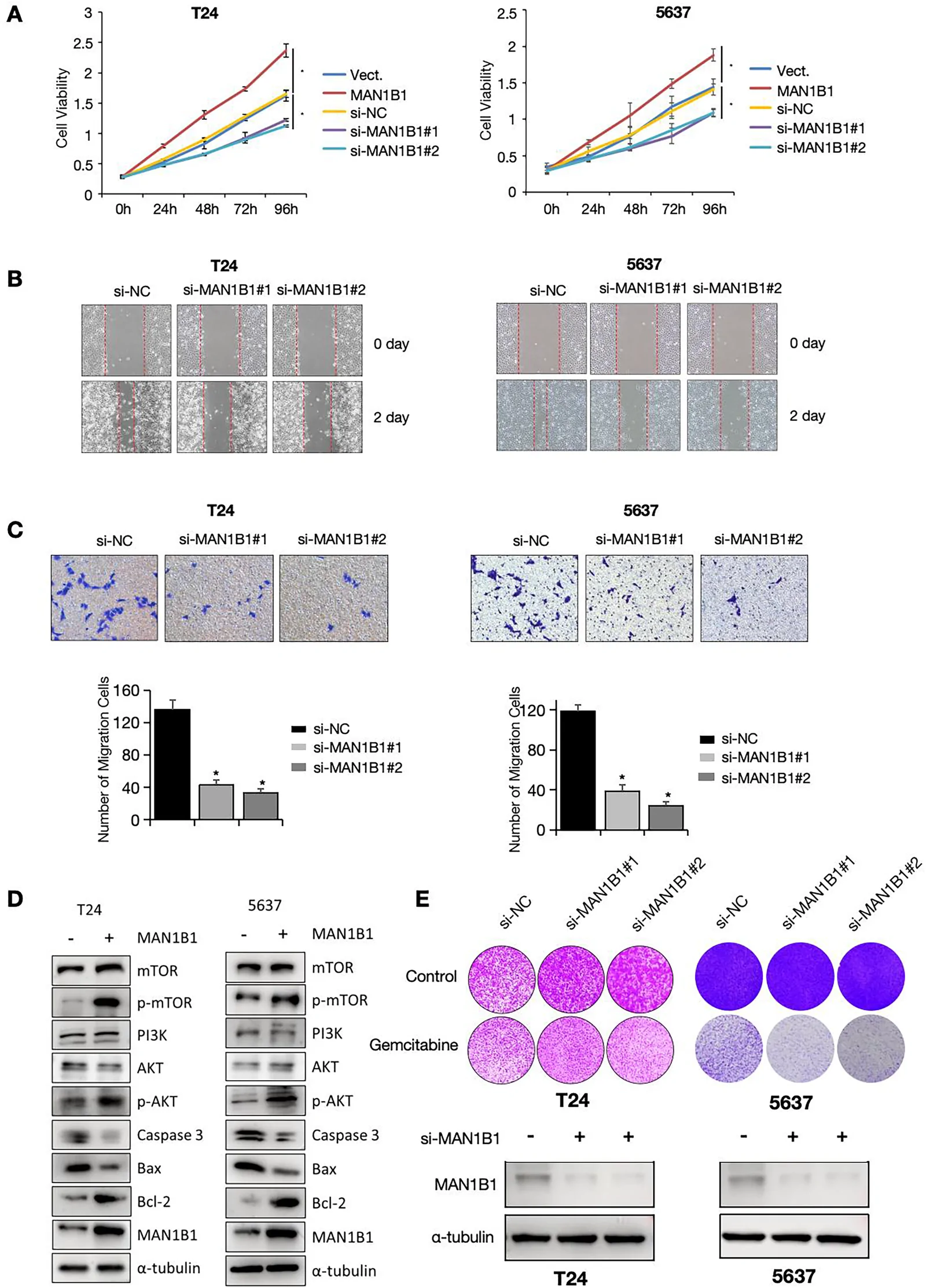
The functional role of MAN1B1 in bladder cancer cell lines. **(A)** Cell Viability Assay. Cell viability of T24 and 5637 cell lines at different time points (0 h, 24 h, 48 h, 72 h, 96 h) following MAN1B1 knockdown or overexpression. **(B)**.Scratch Assay. Cell migration analysis at 0 days and 2 days post-MAN1B1 knockdown. **(C)** Transwell Assay. Assessment of invasive capacity of cells following MAN1B1 knockdown. **(D)** Western Blot Analysis. Detection of the PI3K/AKT/mTOR signaling pathway. **(E)** Colony Formation and Western Blot Assays. Evaluation of gemcitabine sensitivity following MAN1B1 knockdown. *P < 0.05, Student’s t-test.

## Discussion

As a prominent nucleoside derivative, gemcitabine is widely applied as an antineoplastic compound (27). In the urothelial malignancies’ clinical management, this pharmacological agent remarkably reduces postoperative relapse probabilities after endoscopic tumor ablation (28, 29). Although studies on synergistic regimens, prolonged-release formulations, and monotherapy protocols increase gradually, there is still disappointingly limited clinical response to local instillation (30–35). Geriatric demographics and individuals with weakened immune systems reflect evidently decreased therapeutic sensitivity and acquired pharmacological tolerance (18). Therefore, chemoresistance severely affects clinical results, eventually leading to neoplastic relapse and systemic dissemination. Besides, residual tolerant clones often show aggressive oncogenic phenotypes, featured by extensive metastatic spread and accelerated cellular division (36–38). Hence, illustrating complex molecular pathways propelling chemotherapeutic tolerance is still an absolute prerequisite for reinforcing intervention success.

This study has examined MAN1B1’s regulatory role in urothelial carcinoma advancement as well as its possible association with gemcitabine insensitivity. By synergistically applying structural simulations, prognostic evaluations, computational modeling, and *in vitro* empirical assays, our work reveals that this specific protein functions as a critical modulator that governs infiltrative capacity, motility, malignant cell viability, and pharmacological tolerance. Such findings lay a solid foundation for later research on therapeutic utility in urological oncology.

This study examined the GSE190636 transcriptomic repository to isolate transcriptional variations associated with chemotherapeutic tolerance across many temporal phases. As indicated by subsequent functional categorization, these transcripts were profoundly overrepresented within biological cascades that governed leading-edge cellular dynamics, focal adhesion complexes, extracellular scaffold restructuring, and actin cytoskeleton attachment. According to Kyoto Encyclopedia mapping, components had obvious accumulation in the Hippo signaling cascade. Such specific signaling axis exerts strong oncogenic impact across various human malignancies, which propels epithelial-to-mesenchymal changes, cultivates stemness traits, inhibits programmed cell death, and boosts pharmacological insensitivity (39–45). As underscored by previous literature, this cascade plays a critical role in mediating chemotherapeutic evasion. For example, research indicated that hepatic leukemia factor stimulates this exact signaling network to drive ovarian neoplasm advancement and drug tolerance (46). As claimed by Hao et al., upregulation of the JAG-1 polypeptide empowers this cascade to spark Notch communications, thereby diminishing drug-induced apoptotic responses and augmenting chemoresistance (47). Importantly, the foundation for this regulatory axis is transcriptional co-activators YAP and TAZ. Ectopic stimulation across many neoplasms, plus nuclear translocation of YAP, suppresses cytotoxic T lymphocyte-mediated antineoplastic immunity by modulating PD-1 and PD-L1 transcript levels (48). Hence, it is of great scientific value to explore complicated molecular operations that govern this signaling module. By formulating precision medicines directed against these specific molecular nodes, urothelial carcinomas’ immune suppression and pharmacological evasion could be alleviated.

Critical perspectives about prospective ligand-receptor binding dynamics were generated through computational simulations. This study aligned transcriptional variations to respective three-dimensional macromolecular conformations while executing virtual binding assays, so that many polypeptides with strong interactive strengths (i.e. EIF3M, KEAP1, ACLY, KIF20A, EML2, MAN1B1, ADAM8, PMP22, and RIMKLB) were identified. Besides, these specific transcripts’ medical relevance was confirmed through prognostic evaluations. Higher transcript levels of MAN1B1 were profoundly correlated with decreased overall longevity. This indicates its utility as a predictive index for urological malignancies. Although alternative targets such as ADAM8, KIF20A, ACLY, KEAP1, EIF3M, and PMP22 showed directional trends concerning longevity disparities, there was deficient rigorous statistical validation for such correlations. Abundant MAN1B1 production is strongly related to shortened lifespan. Therefore, it is of great translational significance. Previous studies have shown that MAN1B1 has been identified as a harmful predictor and is highly expressed in most malignant tumors. Hepatitis B virus promotes cancer development by increasing the expression of MAN1B1 (49). MiR-125b can regulate cancer formation by targeting the products of the MAN1B1 gene (50). Knockdown of MAN1B1 can reduce the migration and invasion ability of cancer cells in colon cancer (51). In BC, Jie Zhang and his team found that ERK activation can lead to the stabilization of MAN1B1 through HRD1 regulation. Stable MAN1B1 can further promote the glycosylation of CD47, enhance the interaction between CD47 and SIRP -α, and ultimately drive the immune escape of bladder cancer cells (52).

Gene set enrichment profiling offered profound mechanistic insights, demonstrating that abundant MAN1B1 transcripts intimately associate with stimulation of diverse metabolic cascades, encompassing bioenergetics, lipid catabolism, endocrine and micronutrient processing, alongside xenobiotic biotransformation. Such metabolic rewiring constitutes a hallmark of oncogenesis, wherein malignant cells recalibrate their bioenergetic circuits in response to microenvironmental nutrient scarcity and immunosurveillance, thereby fueling expansion and systemic dissemination (53). Conversely, elevated MAN1B1 levels corresponded with marked suppression of immunological cascades, specifically NOD-like receptor communications, natural killer cell cytotoxicity, antigenic peptide handling, and IgA-driven mucosal immunity. Such defensive mechanisms remain absolutely essential for robust antineoplastic surveillance. Consequently, MAN1B1-driven attenuation of host immunity likely facilitates immune escape, subsequently accelerating neoplastic advancement and fostering chemotherapeutic insensitivity.

Based on single-cell RNA sequencing, the specific cellular provenance of MAN1B1 and its contributions to the neoplastic ecosystem were clarified. This transcript was mainly localized in epithelial compartments, suggesting that its biological activities primarily came from malignant cells themselves. Pseudotemporal trajectory mapping revealed that MAN1B1 abundance progressively declined over developmental time, indicating a relationship with distinct malignant phenotypes. As demonstrated by subsequent intercellular crosstalk assessment, MAN1B1-positive epithelial subsets actively participated in Hippo cascade transmissions, extracellular matrix receptor engagements, and AMPK signaling events. These observations are consistent with previous transcriptomic enrichment findings that MAN1B1 not only functions intrinsically within malignant clones, but also restructures matrix-dependent cues and paracrine signaling architectures to coordinate microenvironmental reprogramming.

In silico gene ablation modeling suggested that MAN1B1 participated in assembling immunoregulatory circuitries. Additionally, computational perturbation via scTenifoldKnk uncovered that target transcripts such as TAGAP and TRAF3IP3 underwent profound dysregulation after MAN1B1 elimination. Furthermore, the resulting cooperative expression architecture primarily emphasized positive modulation of T and B lymphocyte receptor cascades, leukocyte-driven defense, cytokine secretion, NF-κB transmissions, together with immunological events like transendothelial leukocyte trafficking. Based on such data, this study validated immunosuppressive trends observed in enrichment profiling, implying that MAN1B1 might disrupt molecular crosstalk between malignant clones and their surrounding defensive milieu to boost immune evasion phenotypes. This study proposes a hypothesis that MAN1B1 not only stimulates cellular division, but also establishes an immunosuppressive niche favoring neoplastic persistence, thereby contributing to urothelial malignancies.

*In vitro* perturbation experiments showed that MAN1B1 knockout was associated with decreased cell survival, delayed wound closure, reduced cell penetration through Transwell membranes, and increased sensitivity to gemcitabine. The Transwell assay performed quantitative cell counting, while the scratch assay provided qualitative supporting evidence based on representative images. Importantly, depletion of MAN1B1 also reduced cell viability, which may be one of the reasons for the changes observed in scratch and Transwell assays. Of course, current research results support the association between MAN1B1 expression and migration or invasion related phenotypes, but cannot prove that MAN1B1 independently regulates intrinsic cell movement or invasion. As verified by immunoblotting assessment, forced MAN1B1 expression evokes the PI3K/AKT/mTOR signaling cascade. This specific molecular axis manages basic biological processes, such as longevity, proliferation, cellular motility, and metabolic homeostasis within oncological contexts (54, 55). As indicated by current literature, activation of this particular cascade can lead to pharmacological insensitivity. As pointed out by Yuge et al., nicotine exposure accelerates neoplastic expansion while triggering acquired chemotherapeutic tolerance via stimulation of the PI3K/AKT/mTOR axis in urothelial models (56). In this experiment, increased MAN1B1 levels greatly elevated protein abundance of PI3K, phosphorylated mTOR, mTOR, Bcl-2, phosphorylated AKT, and AKT, and inhibited pro-apoptotic factors Caspase-3 and Bax.

MAN1B1, as an endo - α - mannosidase, catalyzes the removal of mannose residues from N-linked polysaccharides in the endoplasmic reticulum and cis Golgi apparatus. This enzyme activity regulates protein glycosylation patterns and is involved in quality control of glycoprotein folding and transport. Given the role of protein glycosylation in regulating protein conformation, stability, and signaling ability, we propose that MAN1B1 mediated glycan remodeling may affect one or more components of the PI3K/AKT/mTOR pathway through the following potential mechanisms: MAN1B1 mediated glycan remodeling may affect the glycosylation status of receptor tyrosine kinase (RTK) or its ligands, thereby regulating receptor activation and initiation of PI3K signaling. Abnormal N-linked glycosylation is associated with changes in RTK function in cancer cells; Therefore, changes in MAN1B1 activity also affect the upstream triggering of the PI3K/AKT/mTOR cascade.

There are still some limitations to this study. Firstly, although cytological testing shows that MAN1B1 is associated with drug tolerance and tumor expansion, its physiological significance *in vivo* still needs to be validated through mouse models. Secondly, the use of siRNA targeted knockout cannot completely eliminate off target effects, and specificity should be confirmed by re expressing siRNA resistant constructs. In migration invasion analysis, due to the simultaneous decrease in cell survival rate caused by MAN1B1 knockout, it is difficult to clearly distinguish whether the changes in motility are a direct effect or a secondary result of cell survival; And no standardized EMT markers were examined, so the observed phenotype cannot be directly attributed to EMT reversal. In terms of mechanism research, the activity of the PI3K/AKT/mTOR pathway has not been quantitatively evaluated using phosphorylation to total protein ratios, nor have pathway inhibition or rescue experiments been conducted to demonstrate its causal mediating role. For the enhancement of sensitivity to gemcitabine, there is a lack of IC50 comparison, dose-response curve, and formal joint analysis, and no apoptosis related indicators have been measured, so it is impossible to determine the additive or synergistic effects and their mechanisms. In addition, using only cancer cell lines and lacking a control of normal urinary tract epithelial cells, tumor selectivity cannot be determined. Finally, although the relationship between the enzymatic function of MAN1B1 and glycosylation regulation signal transduction is theoretically established, its specific glycoprotein and oligosaccharide modifications with the PI3K/AKT/mTOR pathway have not yet been identified, and further understanding of the mechanism is needed through glycoproteomics and biochemical analysis.

## Conclusion

To conclude, MAN1B1 is a pivotal modulator that governs gemcitabine insensitivity and urothelial carcinoma advancement. Through the stimulation of PI3K/AKT/mTOR axis, immunological escape, and metabolic rewiring, this specific molecule propels pharmacological tolerance, infiltrative behavior, and malignant cell viability. Therefore, directing pharmacological interventions against MAN1B1 stands for a highly viable clinical method that can strengthen chemotherapeutic responsiveness and refine prognostic trajectories for influenced persons.

## Data Availability

The original contributions presented in the study are included in the article/supplementary material. Further inquiries can be directed to the corresponding author.
